# Independent Paroxysmal Nocturnal Hemoglobinuria and Myelodysplastic Syndrome Clones in a Patient With Complete Bone Marrow Failure

**DOI:** 10.1097/HS9.0000000000000142

**Published:** 2018-09-19

**Authors:** Masayuki Mita, Tsutomu Shichishima, Hideyoshi Noji, Hiroshi Takahashi, Ken-Ichi Nakamura, Takayuki Ikezoe

**Affiliations:** 1 Division of Hematology and Oncology, Shirakawa Kosei General Hospital, Shirakawa, Fukushima, Japan; 2 Department of Hematology, Fukushima Medical University, Fukushima, Japan.

Aplastic anemia (AA), paroxysmal nocturnal hemoglobinuria (PNH), and myelodysplastic syndrome (MDS) are types of acquired bone marrow failure (BMF) syndromes. The coexistence of MDS and PNH as both full-blown disorders is a rare and clinically significant phenomenon. Here, we describe the coexistence of an independent PNH clone and a del(5q) clone in a patient with both disorders.

A 70-year-old woman presented with a 10-year history of coronary spasm, mitral valve regurgitation, and chronic heart failure. In March 2010, she was admitted to the hospital because of shortness of breath and anemia. Bone marrow aspiration showed slight hypocellularity with dysplastic features. She was diagnosed with MDS, refractory cytopenia with multilineage dysplasia, according to the 2008 World Health Organization classification system. She was categorized to have intermediate-1 risk by the International Prognostic Scoring System, and was then treated with methenolone acetate and received a red blood cell (RBC) transfusion. Despite these treatments, the anemia progressively worsened, and she became transfusion-dependent. In August 2011, she was admitted to our hospital complaining of easy fatigability and shortness of breath. Her complete blood count was as follows: hemoglobin, 54 g/L; RBCs, 1.46 × 10^12^/L; white blood cells, 3.7 × 10^9^/L; and platelets, 82 × 10^9^/L. Her serum indicated hemolysis: lactate dehydrogenase (LDH) levels were elevated to 1157 IU/L (normal range, 119–229 IU/L), with haptoglobin levels below 0.10 g/L (normal range, 0.19–1.7 g/L) and a high reticulocyte fraction of 7.0% (normal range, 0.2–2.6%). The direct Coombs test produced a negative result, hemosiderin granules were found in the urine. Bone marrow aspirate showed slight hypocellularity with erythroid hyperplasia and trilineage myelodysplasia, and cytogenetic analysis revealed the abnormal karyotype 46,XX,del(5)(q23q32). The level of Wilms’ tumor 1 (WT1) mRNA in the bone marrow sample was elevated to 990 copy/μg RNA (normal range, <50). Immunohistochemically, several p53-positive cells were present in the bone marrow clot section. These findings demonstrated that apparent intravascular hemolysis and abnormal karyotypes emerged on the background of BMF (Table 1). Flow cytometry of peripheral blood revealed CD55- and CD59-negative erythrocyte proportions of 43.9% and 42.6%, respectively. Moreover, in October 2011, single-color flow cytometric analysis of peripheral blood, using monoclonal antibodies, showed a significant PNH population^1^: 14.8% of erythrocytes and 51.9% of granulocytes tested negative for CD59, while 56.2% of monocytes and 1.6% of lymphocytes tested negative for CD48. These results indicated the presence of cells deficient in glycosylphosphatidylinositol (GPI)-anchored proteins (AP), confirming the diagnosis of PNH.

**Table 1:**
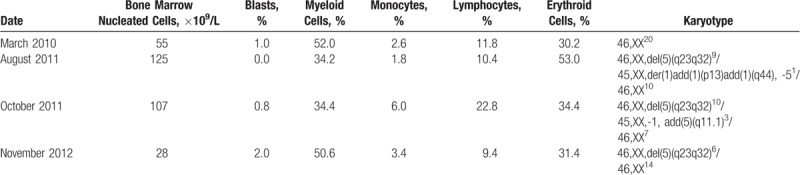
Bone Marrow Differential Cell Counts and Karyotypes

Next, we sought to determine whether the abnormal karyotype arose in the GPI-AP-deficient or GPI-AP-normal granulocytes. To accomplish this, in May 2012 we performed analysis of del(5)(q23q32) with peripheral blood specimens. We isolated 2 different populations (GPI-AP-normal and GPI-AP-deficient granulocytes) and then tested for the loss of *EGR1* and *CSF1R*. We sorted peripheral blood granulocytes into GPI-AP-normal and GPI-AP-deficient populations by flow cytometry,^1^ and then used the *CSF1R* fluorescence in situ hybridization (FISH) probe to detect the *CSF1R* gene located on chromosome band 5q32 and the *EGR1* gene on chromosome band 5q31. FISH analysis showed an absence of 5q31 (*EGR1*) and 5q32 (*CSF1R*) rearrangements. Presumably, the loss of *EGR1* and *CSF1R* was seen on only 1 copy of chromosome 5. Instead, we observed a loss of *EGR1* and *CSF1R* in, respectively, 94.0% and 97.1% of interphase GPI-AP-normal granulocytes, and in 0.0% and 0.1% of GPI-AP-deficient granulocytes. These results demonstrated that del(5)(q23q32) was present in the non-PNH clone but not in the PNH clone.

**Figure 1:**
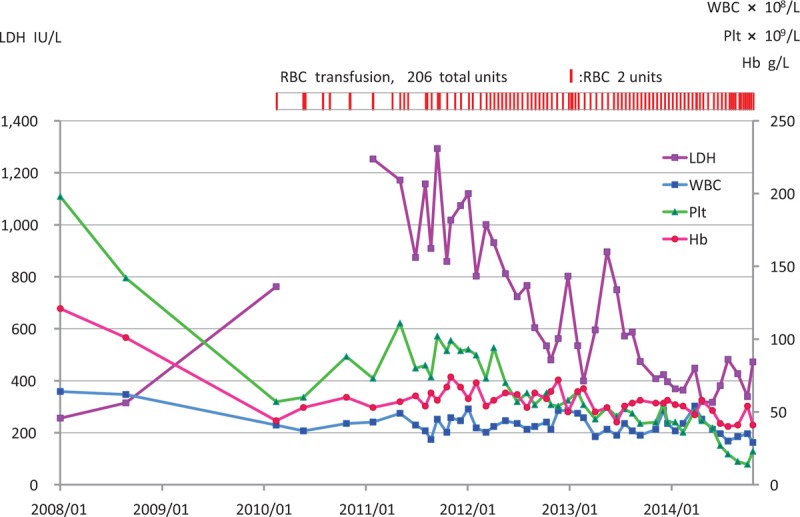
**Time course of LDH level, WBC count, Plt count, and Hb level.** Hb = hemoglobin, LDH = lactate dehydrogenase, Plt = platelet, RBC = red blood cell, WBC = white blood cell.

The patient did not accept treatment with eculizumab. Instead, she finished treatment with methenolone acetate, and received only fortnightly to monthly RBC transfusions. Her serum LDH rose as the intravascular hemolysis progressed and reached a peak of 1293 IU/L in October 2011 (Fig. 1). In October 2012, subsequent single-color flow cytometric analysis of peripheral blood showed a significant PNH population: 21.9% of erythrocytes, 68.6% of granulocytes, 70.5% of monocytes, and 2.0% of lymphocytes. She died of progressive heart failure 39 months after being diagnosed with PNH.

There were 2 significant findings in our case. First, our patient simultaneously had PNH and MDS as full-blown disorders. In studies using high-resolution flow cytometry, approximately 40% to 60% of patients with AA and 2% to 20% of patients with low-risk MDS have been found to have detectable populations of GPI-AP-deficient erythrocytes and granulocytes.^1–6^ The threshold separating subclinical PNH from clinical PNH is reached when the granulocyte clone size is between 20% and 25%, with a corresponding GPI-AP-deficient erythrocyte population of 3% to 5%.^2^ Longitudinal studies have indicated that clonal expansion occurs in 15% to 50% of cases.^2,3^ However, our patient was followed up for more than 10 years after her initial heart disease; the PNH clone might have emerged in September 2008, because hematological data indicated the beginning of anemia, thrombocytopenia, and LDH elevation without any clinical symptoms of hemolysis (Fig. 1). It is reported that there are no differences in the extent of morphological abnormalities between PNH and MDS.^7^ In March 2010, she was diagnosed as having MDS by cell morphology, but flow cytometry for PNH was not performed. In 2011, elevated WT1 mRNA, p53-positive cells, and chromosome abnormalities in the bone marrow all supported the diagnosis of MDS. Clinically, hemolysis and hemoglobinuria are 2 of the major findings in PNH. As shown in Figure 1, the transfusion requirement of this patient was very high, and it increased over the period from October 2011 to October 2012, when the GPI-negative granulocytes increased from 51.9% to 68.6%. The patient received 44 units of red cells, which is higher than one would expect in MDS, and the high transfusion requirement can be attributed in large part to hemolytic PNH. In 2010, the patient likely had anemia, thrombocytopenia, and a hypocellular marrow; she probably already had mild AA, but by the time she came to our attention, her marrow was being re-populated by the PNH clone and by the del(5q) clone, thus masking AA.

Second, we then demonstrated that the PNH clone and the del(5q) clone found in MDS arose independently, as evidenced by our observation of del(5)(q23q32) in GPI-AP-normal granulocytes fractions but not in GPI-AP-deficient granulocytes. PIG-A mutations, or rare PIG-T mutations, are the causes of disrupted GPI-AP synthesis, but alone they are not sufficient for the expansion of a PNH clone. The process behind the clonal expansion of PIG-A mutated stem cells in PNH patients is not fully understood.^7–10^ Several hypotheses have been proposed, which are not mutually exclusive: (1) PNH cells escape immune attack, leading to the proliferation of PNH clones to gain an advantage. (2) Stem cells with a mutated *PIG-A* gene are relatively resistant to apoptosis. (3) PNH cells acquire additional mutations and a selective advantage. Moreover, according to recent genetic insights into the mechanisms of clonal evolution in PNH, 4 models for the sequence of events have been proposed.^10^ (a) *PIG-A* is the only gene mutated at the clonal level (52.6%). (b) Additional myeloid mutations arise in a *PIG-A* mutated clone (10.5%). (c) A secondary *PIG-A* mutated subclone arises within the primary myeloid mutated clone (31.6%). (d) Both myeloid and *PIG-A* mutated clones independently coexist (5.3%). Our case seems to follow mechanism “(d),” which is a rare category.

In conclusion, patients diagnosed with MDS must be followed up, with bone marrow analyses and PNH clone measurements, to detect the development of other hematological diseases. Future studies may resolve the mechanisms of clonal expansion in PNH.
